# Royal College of Surgeons in London

**Published:** 1833-10

**Authors:** 


### II. ROYAL COLLEGE OF SURGEONS IN LONDON.

The Council of the College of Surgeons has lately published a statement relating to its museum, library, and finances, sufficiently interesting to induce us to reprint a considerable part.

"In pursuance of a Resolution of the Council, on the 29th of January last, "*That an exposition of the state of the College be from time to time made to its Members*," the Council publish the following STATEMENT:

"The Corporation of Surgeons, established as a distinct body, by Act of Parliament, in the year 1745, having become dissolved in consequence of an accidental informality in their proceedings, the present Royal College of Surgeons was founded, in the year 1800, by his Majesty King George the Third, for the advancement of surgery, for the examination of surgeons in the army and navy, and of other individuals who might wish to engage in the surgical profession.

"The repute in which the corporation was held appears to have been so limited, that a large proportion of the practitioners throughout the kingdom had undergone no examination, and had not even a nominal connexion with it. The inheritance derived by the college from the corporation was as follows: 10,135*l.* 7s. 5d.

3 per cent. consols; 1233*l.* 15s. 0d. due from the city of London for the hall in the Old Bailey; 2862*l.* 16s. 7d. in exchequer bills and at the banker's; the house in Lincoln's Inn Fields, which then occupied one half of the site of the present premises; and a rent-charge of 16*l.* per annum on premises in Snow Hill, bequeathed by Mr. Gale for the endowment of a professorship of anatomy. There were no certain funds of any other description, for defraying the expense of maintaining the great national collection purchased by parliament of the executors of Mr. John Hunter, and which the college now holds in trust for the public.

"When the college received its charter from the crown, it derived no assistance of any kind from the other branches of the legislature. The charter was simply permissive, allowing the court of examiners to examine those who might voluntarily present themselves, but giving them no legal authority whatever, to compel practitioners in surgery to obtain their diploma, nor to prosecute those who took upon themselves to practise without it. The college, therefore, possessing no other influence than that of opinion, was left to rest altogether on its own character. Under these circumstances, it would never have advanced to its present state of prosperity, if it had failed to obtain the confidence of the profession and the public; and the best proof that it has succeeded in this object, is to be found in the increased and increasing number of the members. In the first two years after the establishment of the college, the diploma was granted to three hundred, and in the last two years to not fewer than 770 members.

"Although the college derives an important accession to its scientific character, from the possession of the Hunterian collection, its preservation and public uses have been a source of great expense to the institution.

"The sum of 27,500*l.* obtained from parliament, having been insufficient for building the museum, an addition, amounting to nearly as much, was supplied from the funds of the college. But, independently of what has been laid out on the building of the museum, about 36,000*l.* have been expended on its contents. The council have always regarded the charge of the collection as one of the most important trusts of the college; and they believe they have best performed their duty, by sparing no expense which might tend to make it as complete as possible.

"Thirty lectures are delivered annually in the theatre of the college by two professors appointed by the council. To these the members of the college are admitted by right, and the senior students of the metropolitan hospitals by courtesy.

"The library, collected within the last six years, and comprising the most valuable works in medicine and surgery, as well as in general science, is open to the members of the profession and other scientific persons, on the most liberal conditions.

"Notwithstanding the large demands made upon the funds of the college by the maintenance of the collection, by the formation of the library, and by the management of the general business of the institution, the annual expenditure has hitherto been kept within the limits of its income, so that a considerable funded property has gradually accumulated, which is now at the disposal of the council, to be applied, as opportunities may occur, for the advancement of the sciences connected with surgery.

"*Finances.* The receipts of the college are, and ever have been, chiefly derived from the sum paid for the diploma; and, inasmuch as there is no well defined law obliging a student to seek this distinction previously to his commencing practice as a surgeon, the income from this source is uncertain. The expenses of the college and library, and especially those entailed by the possession of the Hunterian collection, have been very considerable. It has therefore been a great object with the council to realize such a permanent income as may be sufficient to meet this unavoidable expenditure, in the event of their usual annual resources being materially diminished."

